# Habitat filtering, not dispersal limitation, drives ant and termite community assembly along a tropical forest regeneration gradient

**DOI:** 10.1007/s00442-026-05875-9

**Published:** 2026-03-15

**Authors:** Nina Grella, David A. Donoso, Jörg Müller, Ana Falconí-López, Annika Busse, Peter Kriegel, Marcel Püls, Dominik Rabl, Sebastian Seibold, Heike Feldhaar

**Affiliations:** 1 Animal Population Ecology, Bayreuth Center for Ecology and Environmental Research (BayCEER), University of Bayreuth, 95440 Bayreuth, Germany; 2 Grupo de Investigación en Ecología y Evolución en los Trópicos EETROP, Universidad de Las Américas, Redondel del Ciclista, Antigua Vía a Nayón, Quito, Ecuador; 3 Field Station Fabrikschleichach, Chair of Conservation Biology & Forest Ecology, Biocenter, University of Würzburg, Glashüttenstr. 5, 96181 Rauhenebrach, Germany; 4 Bavarian Forest National Park, Freyunger Str. 2, 94481 Grafenau, Germany; 5 Wilderness Area Königsbrücker Heide, Königsbrück, Germany; 6 Applied Ecology, Department of Biology, Phillips University Marburg, Karl-Von-Frisch-Straße 8, 35043 Marburg, Germany; 7 ForestZoology, TU Dresden University of Technology, Tharandt, Germany

**Keywords:** Alates, Assembly rules, Chocó, Chronosequence, Reassembly, Secondary succession, Social insects

## Abstract

Regenerating forests comprise a significant proportion of tropical forest ecosystems, yet species assembly mechanisms after anthropogenic disturbances remain poorly understood. Locally established ant communities follow predictable paths along forest regeneration gradients, but whether this results from dispersal limitation or habitat filtering is unclear. Social insects, with highly mobile dispersing reproductives (alates) but sessile colony stages, provide a window to disentangle these mechanisms. We compared assemblages of dispersing alates to workers from established colonies of ants and termites along a chronosequence in the Chocó lowland tropical forest, Ecuador. Our study area comprises a regeneration gradient where agricultural land, regenerating forests (1–37 years old), and old-growth forests are interspersed across a 200-km^2^ landscape mosaic with short distances among land-use types. Alate assemblages of both taxa were independent of forest age but more similar in spatially closer plots. Worker ant assemblages were more similar at similar succession stages and elevations. Termite worker assemblages were more similar at similar elevations but more dissimilar in spatially closer plots. These results indicate that alates can disperse across all succession stages and elevations within our study area, but not all species successfully establish or persist everywhere. For ants, colony distribution is determined by habitat filters associated with forest age and elevation. For termites, colony distribution is influenced by elevation-related habitat filtering. Importantly, we found increasing species diversity with forest age for both taxa, underscoring the importance of advanced natural forest regeneration and old-growth forest conservation for maintaining diverse social insect communities.

**Supplementary Information:**

The online version contains supplementary material available at 10.1007/s00442-026-05875-9.

## Introduction

Understanding species assembly rules in recovering forests is of particular importance as they cover large areas globally (FAO 2020) and offer the potential to mitigate species loss during ongoing deforestation (Chazdon et al. 2009). However, unraveling the fundamental mechanisms that govern community assembly remains a central challenge in contemporary ecological theory (Vellend 2010). Among the various proposed concepts, two key mechanisms are dispersal limitation and habitat filtering (Belyea & Lancaster 1999). The dispersal limitation paradigm emphasizes the importance of dispersal between patches. Following this paradigm, differing species compositions in a landscape are explained by the species’ inability to reach suitable habitats due to limited dispersal mechanisms (Leibold et al. 2004). In contrast, the habitat filtering paradigm emphasizes the role of habitat heterogeneity caused by environmental differences in structuring communities, where environmental conditions determine if species can establish and persist in a local patch (Kraft et al. 2015). It assumes a moderate dispersal rate which allows species to reach suitable patches but stresses the importance of niche separation caused by local biotic interactions and abiotic conditions (Leibold et al. 2004). The terms ‘environmental conditions’ and ‘environmental filtering’ often refer to abiotic factors such as temperature and humidity only. Here, we use the term ‘habitat filter’ to emphasize that besides abiotic conditions, also biotic interactions such as mutualism or competition can have an influence on community assembly. However, the relative importance of dispersal limitation and habitat filtering in structuring ecological communities continues to be debated, and both mechanisms could potentially produce and explain the same observed pattern of local species distributions.

In this study, we aim to disentangle the effects of dispersal limitation and habitat filtering for the assembly of ant and termite communities in a tropical forest regeneration gradient. These social insects provide an ideal model system because they exhibit distinct life stages which might be affected by distinct assembly mechanisms: a dispersing phase with enhanced mobility (winged alates) and a colonial phase (workers from established colonies) more focused on local establishment, allowing us to separate dispersal processes from habitat filtering during establishment. We studied communities along a chronosequence ranging from agricultural land, regenerating forest of different ages, to old-growth rainforest. This design represents an environmental gradient with differing biotic and abiotic conditions (Escobar et al. 2025; Newell et al. 2025). As our study is situated in the Chocó–Darien Global Ecoregion (CGE), which is among the top 25 biodiversity hotspots for conservation worldwide (Myers et al. 2000), our study area provides a crucial context for evaluating the ecological consequences of land-use transformation (Escobar et al. 2025).

Former investigations in our study area have shown that assemblages of ant workers emanating from established colonies differ between agricultural land, regenerating forest and old-growth forest (Hoenle et al. 2022, 2023). Specifically, forest regeneration time, elevation and land-use legacy have been identified as drivers for species composition (Hoenle et al. 2022, 2023). The sensitivity of ant assemblages to anthropogenic disturbances via deforestation or land conversion has also been extensively documented in tropical systems worldwide, with studies demonstrating shifts in species richness, functional diversity, and community composition following habitat alteration (Dunn 2004; Bihn et al. 2008; Neves et al. 2010; Schmidt et al. 2013; Staab et al. 2014; Gomes et al. 2014; Hethcoat et al. 2019). Based on these outcomes alone, environmental conditions appear to be central in shaping the distribution of ants in this landscape, strengthening the habitat filtering paradigm as pivotal mechanism in explaining ant assemblages. Studies of termite communities have documented similar patterns, showing that colony occurrence responds to anthropogenic disturbances across various tropical systems through changes in species richness and altered community composition (Ackerman et al. 2009; de Paula et al. 2016; Duran-Bautista et al. 2020, 2024; Castro et al. 2021). However, these previous studies examined established colonies and therefore cannot distinguish whether observed community differences arise from dispersal limitation preventing colonists from reaching certain habitats, or from habitat filtering that acts after dispersal has occurred.

To disentangle these two mechanisms, we investigate ant and termite communities one step before colony founding: during the dispersal of winged reproductives (so-called alates). In their mobile life stage, alates of many social insect species leave their natal colony for nuptial flights to disperse into the surrounding landscape for mating and the subsequent establishment of a new colony (Hartke & Baer 2011; Hakala et al. 2019; Donoso et al. 2022; Uquillas et al. 2025). By comparing the assemblages of dispersing alates with those of workers of established colonies, we can determine at which life stage community filtering occurs. We test for the influence of spatial distance (geographic coordinates of plot location), forest age, and elevation on the community composition of both dispersing ant and termite alates and foraging workers from established colonies. Forest age is our primary variable of interest as it represents the forest regeneration gradient and the associated changes in habitat conditions. Elevation is included as an additional habitat filter because topographic gradients have been shown to structure social insect communities independently of forest age in tropical and subtropical systems (Gathorne-Hardy et al. 2001; Palin et al. 2011; Staab et al. 2014; Nunes et al. 2017; Hethcoat et al. 2019; Leahy et al. 2024). By including both forest age and elevation as predictors, we can distinguish between habitat filtering associated specifically with forest regeneration versus other environmental gradients that may operate simultaneously.

Our analytical approach considers different scenarios to test our primary hypothesis that forest regeneration drives community assembly, while also examining elevation and spatial distance as additional factors that may structure communities. First, we examine whether dispersal limitation structures alate communities. If dispersal is not limited, alate  assemblages should be similar throughout our study area regardless of the spatial distance between sampling plots. This would indicate that alates can effectively disperse across the landscape and reach all available habitats. However, the study area encompasses distances that exceed typical dispersal ranges documented for many species of ants (Helms 2018), which are considered better flyers than termites. Thus, we may instead observe that alate communities become increasingly dissimilar with increasing spatial distance. Such a spatial pattern would suggest regional dispersal limitation, where geographic distance prevents alates from reaching distant patches, independent of habitat characteristics. Beyond dispersal limitation, habitat characteristics could also filter alate communities during their dispersal phase. Our study area consists of a small-scale mosaic of different land uses and forest ages with short distances between habitat types (Escobar et al. 2025), which should allow alates to reach plots across the full range of forest ages and elevations in our chronosequence. If alate communities differ among forest ages or elevations, this would indicate that habitat filters influence assemblages already during the dispersal phase. Such filtering could occur through behavioral habitat selection by alates responding to environmental cues, through environmental conditions (temperature, humidity, wind patterns) that vary with forest age and elevation affecting flight capacity or survival, or through the spatial distribution of source colonies already reflecting habitat preferences that determine which alates are available to disperse into different areas.

Second, if alate assemblages show similar composition across forest ages and elevations but worker assemblages differ markedly among these habitat types, this would indicate that habitat filtering operates primarily after dispersal. Such a pattern would suggest that microhabitat requirements for colony establishment, resource availability for colony maintenance, or biotic interactions affecting colony survival vary across the forest age gradient or elevational range.

Third, if both alate and worker communities differ among forest ages and elevations, this would demonstrate that habitat filtering influences community composition during the dispersal phase itself, in addition to habitat filtering that may occur post-establishment.

Finally, we acknowledge an alternative scenario where worker communities show no strong association with forest age and elevation, despite differences in alate communities. Although this scenario seems less likely for ants given previous documentation of ant community responses to forest age in our study system (Hoenle et al. 2022, 2023), it would suggest that particular habitat characteristics are less important for colony establishment and persistence than for influencing alate dispersal patterns.

## Methods

### Study sites and plot design

**Fig. 1:**
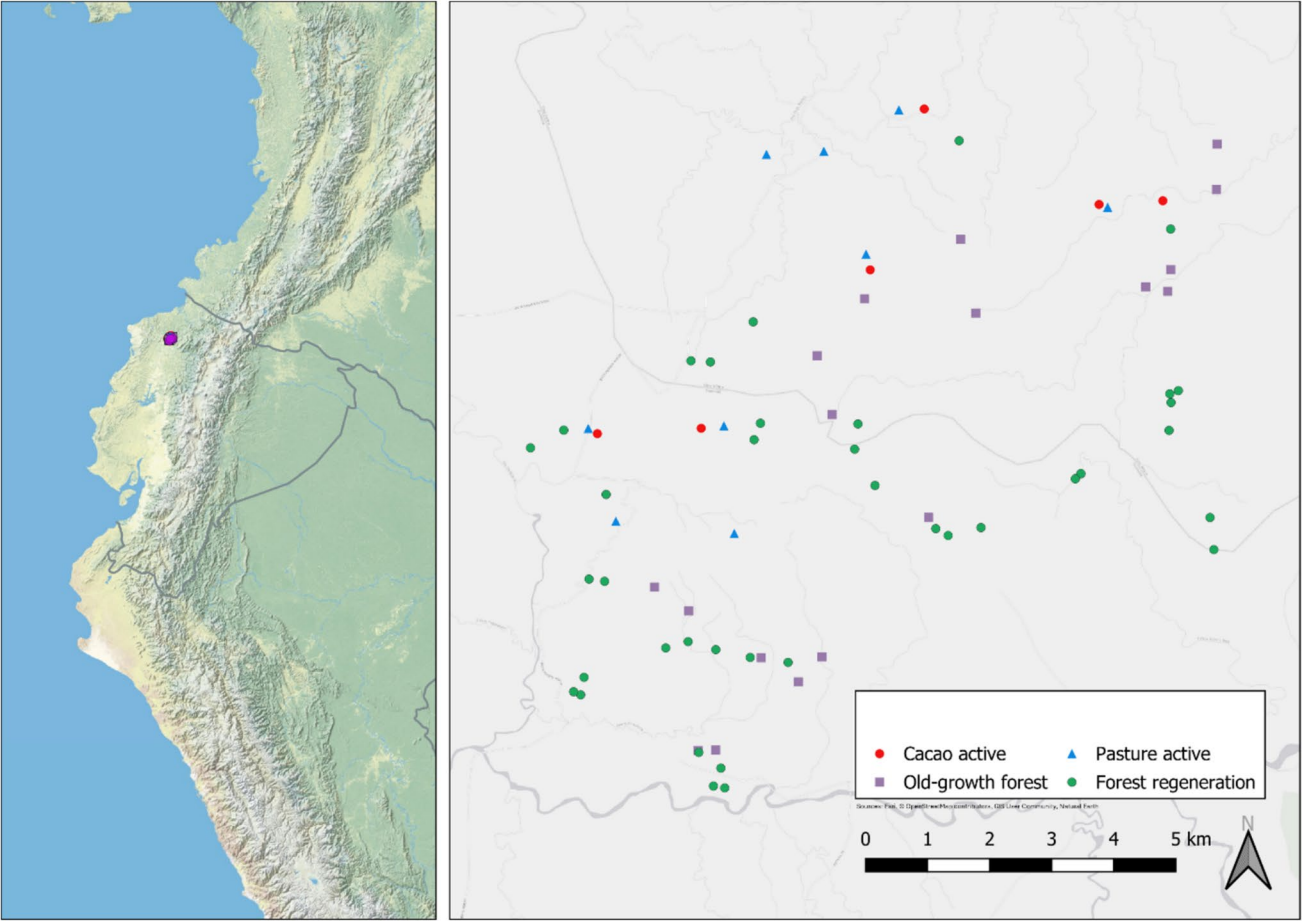
Distribution of the study plots in the reserves Río Canandé and Tesoro Escondido in the Chocó rainforest in north-western Ecuador. Symbols indicate different habitats and land-use legacies of the study plots

Our study area is located in the tropical lowland Chocó rainforest in north-west Ecuador (province Esmeraldas) in the two reserves Reserva Río Canandé and Reserva Tesoro Escondido. 62 plots were selected in the framework of the REASSEMBLY research unit (www.reassembly.de). Plots are located across a 200-km^2^ area and comprise each 50 × 50 m in forest plots (old-growth forest and regenerating forests) and 16 × 16 m in agricultural plots. The landscape in our study area is characterized by a small-scale mosaic of heterogeneous environment encompassing old-growth forests, regenerating forests of various successional stages, human settlements, and agricultural land with short distances among these land-use types. The distance of each plot to the nearest old-growth plot for example averages 59 m (SD ± 46) and forest cover is generally high with an average of 74% (SD ± 2.8) within a 1-km radius of each plot (Escobar et al. 2025). Our study sites consisted of a regeneration gradient ranging from agriculture (pastures and cacao plantations) to regenerating forests (former pastures and cacao plantations) to old-growth forests. Time since abandonment (= forest age) of regenerating forests ranged from 1 to 37 years and plot elevations from 128 to 615 m. The minimum distance between plots of the same type was 184 m, while the largest was 14 km. Between different plot types, the minimum distance between two plots was over 250 m. Within-type inter-plot distances averaged 4.6 ± 1.1 km for active pastures, 5.2 ± 1.1 km for active cacao plantations, 5.3 ± 0.7 km for regenerating pastures, 5.5 ± 0.7 km for regenerating cacao, and 5.5 ± 0.77 km for old-growth forest (Escobar et al. 2025). The distribution of the plots across the landscape is given in Fig. 1. The regeneration gradient reflects a microclimatic gradient with more extreme temperatures and higher variations of humidity in agricultural lands and young regenerating forests compared to older regenerating and old-growth forests (Newell et al. 2025). These differences in microclimate potentially act as a habitat filter on plants and animals including the focus insect groups of this study. Further details on the study sites and the plot design of the Reassembly plots are described by Escobar et al. (2025) and climatic conditions along the regeneration gradient by Newell et al. (2025). Some plots of our study are not in the final plot selection of the REASSEMBLY project because plot selection was finalized in 2022 (see details below).

### Sampling

Our data consists of two datasets. The first dataset comprises dispersing ant and termite alates that were collected during nuptial flights. The second dataset comprises workers of both taxa, where specimens were collected directly from their nests or during foraging.

The first dataset of dispersing ant and termite alates is a subset of data on flying insects which was published by Müller et al. (2023). The collection of all flying insects using light traps was conducted on 44 plots in October and November 2021. These plots comprise 10 agricultural plots (5 pastures, 5 cacao plantations), 13 regenerating pastures with regeneration times ranging from 4 to 34 years, 10 regenerating cacao plantations with regeneration times ranging from 4 to 25 years, and 11 old-growth forests. From 44 plots, 38 were in the final Reassembly plot selection, whereas 6 plots were sampled additionally (see Müller et al. (2023) for a plot map). Although ants and termites are usually not the target taxa of light traps, studies have demonstrated that light traps are suitable for capturing flying ant and termite alates, and that most alates collected by light traps are females (Basset et al. 2020); hence, we extracted the data of ants and termite alates for our analysis. The trapping methods and the metabarcoding approach for species identification are described in Müller et al. (2023). In short, an autonomous light trap was used for one night per plot for 8 h avoiding full moon phase. It was installed at 2-m height in cleared vegetation-free surrounding. To attract insects after dusk, a LED light was used, which is optimized for insect sampling (LepiLED Mini Switch 0.65, UV-mode switched off, Brehm, Jena, Germany). The trap included a plexiglass roof and a funnel leading insects into chloroform. After collection, all insects were dry-stored in a freezer and filtered by size. Laboratory and bioinformatic pipelines for species identification were followed as described in Hausmann et al. (2020) to sequence the CO1-5P (mitochondrial cytochrome oxidase 1) gene of the collected insect bulk samples. This pipeline resulted in a list of taxa occurring on each plot. We did not assign species names to the taxa as many BINs (Barcode Index Numbers) were not clearly assigned to a species name in the BOLD database. We rather refer to the taxa of this dataset and of the worker dataset as OTUs (operational taxonomic units) to keep it consistent within our study.

Foraging and nest-dwelling workers were collected in three sampling seasons in 2022 and 2023. In the first season, from February to April 2022, we sampled all 62 Reassembly plots and two additional plots (one regenerating pasture and one cacao plantation). The 64 plots comprise 12 agricultural plots (6 pastures, 6 cacao plantations), 17 regenerating pastures, 18 regenerating cacao plantations, and 17 old-growth forests. Regeneration times of both former pastures and cacao plantations range from 1 to 37 years. We collected ants and termites with the use of Winkler traps, by hand sampling of foraging ants and termites on the ground and from tree trunks at breast height, as described by Hoenle et al. (2022). In doing so, we captured ant and termite communities of different strata including leaf litter dwelling (Winkler traps), epigeal (ground transects), and parts of tree dwelling (tree trunks) communities. In addition to the three methods used by Hoenle et al. (2022), we used methods to capture dead wood-dwelling ants and termites. In the first field season, we opened five naturally occurring dead wood pieces per plot (mean per plot: 3.16 ± 1.24, n = 193, 61 plots) that were in contact with the ground (logs and stumps) and collected nesting ants and termites by opening the dead wood. In the second sampling season, from August to October 2022, we used an additional baiting approach on the 62 Reassembly plots where we collected wood-dwelling ants and termites with dead wood baits from five different tree species. We placed one wood piece per plot from the tree species *Trema micrantha* (Sapanillo), *Theobroma cacao* (Cacao), *Inga sp*. (Guaba), *Triplaris cumingiana* (Fernán Sánchez), and *Hieronyma chocoensis* (Mascarey) each. Wood pieces had a diameter ranging from 6 to 12 cm and a length of 50 cm. In the third field season, after 6 months (February–March 2023), we retrieved the five wood pieces from each plot and placed them in emergence chambers. These emergence chambers consisted of mesh tubes made of white fabric used for insect nets (1 mm double-thread netting, bioform.de; Model A110e) and a 50-ml sampling tube filled with ethanol attached at the bottom. Insects emerging from the wood pieces and falling into the ethanol were collected for the following 6 months (February–August).

Sampled ant and termite workers were identified with a combination of morphological analyses and a DNA barcoding approach. First, we identified samples to genus level if possible, using taxonomic literature (Bolton 1994; Constantino 2002) and subsequently separated them into morphospecies. For every sampling method from every plot, we prepared one specimen of each morphospecies for the barcoding of the CO1-5P gene by cutting off one leg of ants and the heads of termites. The dissected tissues were sent to the Canadian Centre for DNA Barcoding (University of Guelph, Canada) for DNA isolation and sequencing. DNA sequences were uploaded to the BOLD (Barcode of Life Data System) database. BOLD creates BIN clusters of sequences that have been shown to be in high concordance with species and can be used for species identification (Ratnasingham & Hebert 2013). Based on these BINs, phylogenetic trees, and additional morphological analyses based on taxonomic literature (as cited in Hoenle et al. 2022), we made our final species identifications. These resulted in a list of taxa for each plot.

### Statistical analyses

We performed statistical analyses separately for both taxa and both datasets using R version 4.4.1 (R Core Team 2023) due to the methodological differences in sampling protocols, sampling intensity, and temporal collection periods between caste types. The sampling of workers from established colonies focused on individuals on the ground or low on tree trunks, thus likely not capturing arboreal ant species. In contrast, the light traps likely captured dispersing alates from ground nesting as well as arboreal species. In addition, worker activity of established colonies should be less variable over time than flight activity of alates, as not all species disperse at the same time and some do not perform nuptial flights at all (Kaspari et al. 2001a, b). However, by analyzing alates and workers separately, we can compare the effects of the selected predictors (regeneration age, elevation, space) on the patterns of the respective communities.

Considering diversity analyses, it is often criticized that species richness does not consider abundance and sampling effort (Gotelli & Colwell 2001). For our data, a standardization considering sampling effort was not possible due to the difficulties of estimating social insect abundance (as discussed by Basset et al., 2023), especially when using a metabarcoding approach where specimens are homogenized after collection. Hence, we used incidence data for the first Hill number q0 (Hill 1973), which is the number of observed species to quantify OTU diversity. We calculated the number of observed OTUs, using the ‘specnumber’ function from the ‘vegan’ package (Oksanen et al. 2022).

For exploring the relation of the number of observed OTUs with forest age and elevation, we performed generalized linear models (GLM) choosing a negative binomial distribution with the ‘glm.nb’ function of the ‘MASS’ package (Ripley et al. 2002). We checked the distribution of residuals using the DHARMa package (Hartig 2024). For this and the following analyses, we set the age of old-growth forests to 55 years to enable inclusion of old-growth forest data. Predictions have shown that tree species richness recovers to 90% in comparison to old growth already after 55 years in our study area (Escobar et al. 2025). We chose this threshold because we assume that a similar tree species richness to old-growth forest would provide a similar resource availability and diversity for foraging (e.g., extrafloral nectaries, seeds, and arthropod prey associated with specific tree species) and nesting (e.g., leaf litter quality and deadwood characteristics).

For the identification of the factors influencing species assemblages between plots, we used a multiple regression on distance matrices (MRM) using the ‘MRM’ function of the ‘ecodist’ package. We made one model for each taxon and caste combination (ant alates, ant workers, termite alates, termite workers) resulting in four multiple regression models. As input, we computed a distance matrix of the Jaccard dissimilarity index with the ‘vegdist’ function of the ‘vegan’ package based on the OTU incidence data on the plots as well as Euclidian distance matrices between plots for forest age, elevation, and space (coordinates of longitude and latitude) with the ‘dist’ function. Afterwards, all matrices were standardized using the ‘decostand’ function. The predictors of these models can indicate toward the presence of dispersal limitation and habitat filtering. When the species compositions of alates differ depending on forest age, elevation, and/or space, we expect a dispersal limitation between plots caused by parameters associated with forest age, elevation, and/or space (plot location). If none of these assemblages differ depending on these predictors, then we expect that alates are not dispersal limited among plots. The same applies to the habitat filtering mechanism for the worker models. When the species composition of workers differs depending on forest age, elevation, and/or space, we expect habitat filtering for certain OTUs induced by parameters associated with forest age, elevation, and/or space, while no effect would suggest there is no strong habitat filter resulting in similar communities between plots. In addition to these models, we used NMDS (non-metric multidimensional scaling) for the visualization of OTU composition along the regeneration gradient with the ‘metaMDS’ function of the ‘vegan’ package by grouping all plots into four categories representing the different land uses with differing habitat conditions in our study area (agricultural land, early regenerating forests (1–20 years), late regenerating forests (21–37 years), and old-growth forest).

## Results

### General results and diversity

**Fig. 2:**
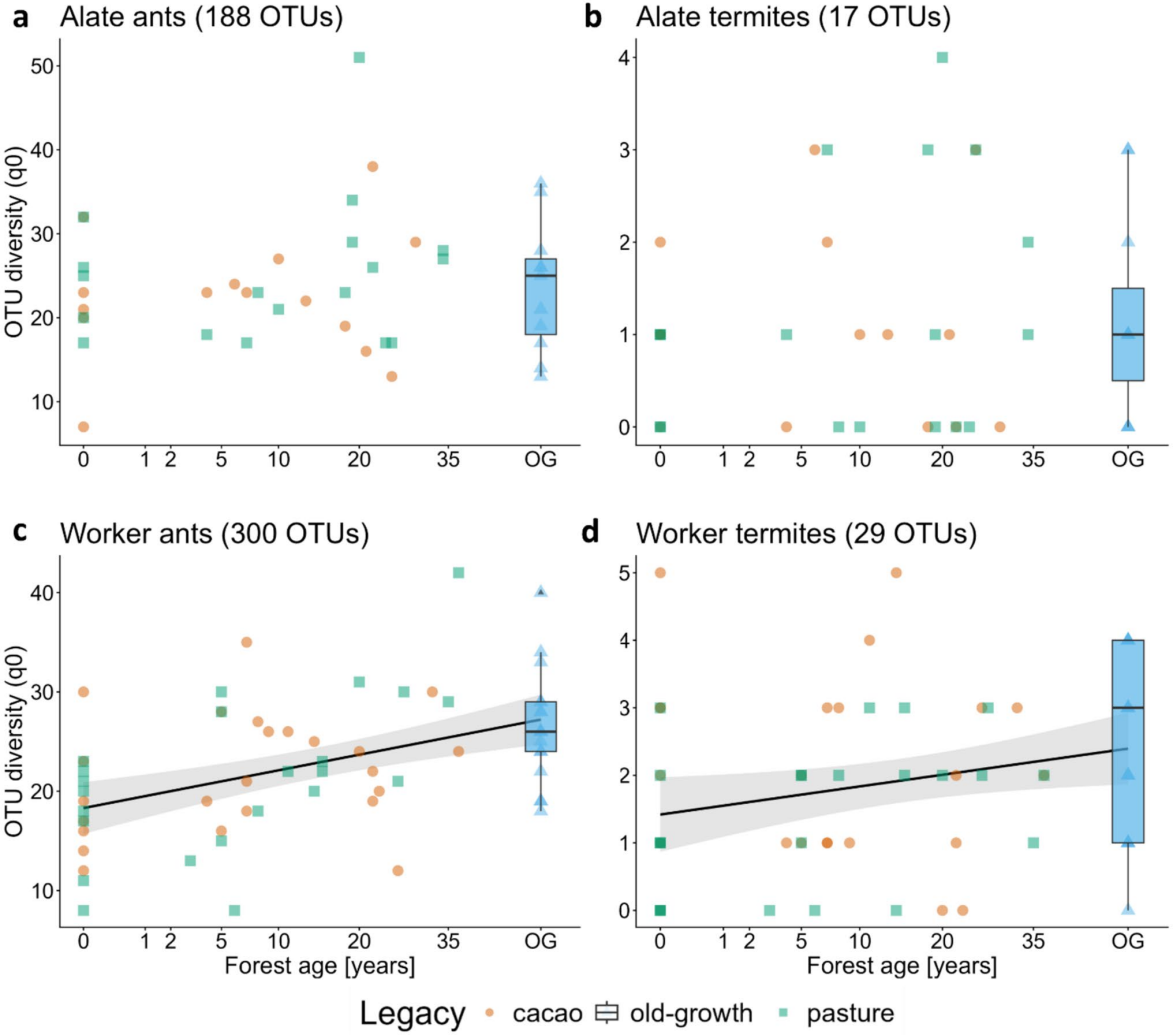
Regeneration trajectories of ant (**a, c**) and termite (**b, d**) diversity (number of observed OTUs, q0) along a tropical forest regeneration gradient. Workers (**c, d**) originate from manual sampling of nest-dwelling or foraging specimen, while alates (a, b) were captured during nuptial flights using light traps. Land-use legacy of active and former pastures and cacao plantations are highlighted by color and shape. Their trajectories are predicted by a linear model with a 95% confidence interval. Solid lines indicate a significant increase in diversity from all plots with forest age according to generalized linear models (Table 1). Old-growth forest (OG) is plotted without forest age for comparison with the regenerating plots

Using the light trap approach for capturing alates, we successfully identified 188 ant OTUs and 17 termite OTUs. From the worker caste, we identified 300 ant OTUs and 29 termite OTUs (Fig. 2). For ants, this slightly exceeds the number of species found in a previous study by Hoenle et al. (2022) in the region (284 morphospecies). For termites, little is known about the local diversity. The alate diversity of both taxa was not influenced by forest age or elevation (Fig. 2a,b; Table 1), while the diversity of worker ants and termites increased with forest age (Fig. 2c,d; Table 1). Worker ants were found in all strata (leaf litter, forest floor, tree trunks, dead wood), and common genera such as *Pheidole*, *Wasmannia*, *Crematogaster*, and *Solenopsis* were found in more than 50 of the 64 sampled plots (Fig. S1 in supplemental information). In contrast, termite workers were observed infrequently on the forest floor and tree trunks, with only three occurrences documented in each of these environments. The majority of termite worker specimens were collected from large pieces of naturally occurring dead wood or in experimental wood baits. The most frequently observed termite genera by plot were *Cylindrotermes* and *Nasutitermes* (Fig. S2 in supplemental information), while the other genera were found in less than 18% of plots.

**Table 1:** Generalized linear models (GLM) of alate and worker ant and termite diversity (number of observed species q0) with forest age and elevation as predictors. Significant p values are highlighted in bold

	Estimate	95% CI	*p* value
Alate ants			
Intercept	3.03	0.14	** < 2e**^**−15**^
Forest age	0.0002	0.002	0.93
Elevation	0.0004	0.0004	0.3
Worker ants			
Intercept	2.98	0.11	** < 2e**^**−16**^
Forest age	0.006	0.002	**7e**^**−5**^
Elevation	8e^−6^	0.0003	0.98
Alate termites			
Intercept	0.29	0.42	0.49
Forest age	0.006	0.008	0.46
Elevation	-0.0009	0.001	0.49
Worker termites			
Intercept	0.68	0.3	**0.02**
Forest age	0.009	0.004	**0.03**
Elevation	-0.0007	0.0008	0.38

### Species composition

**Fig. 3:**
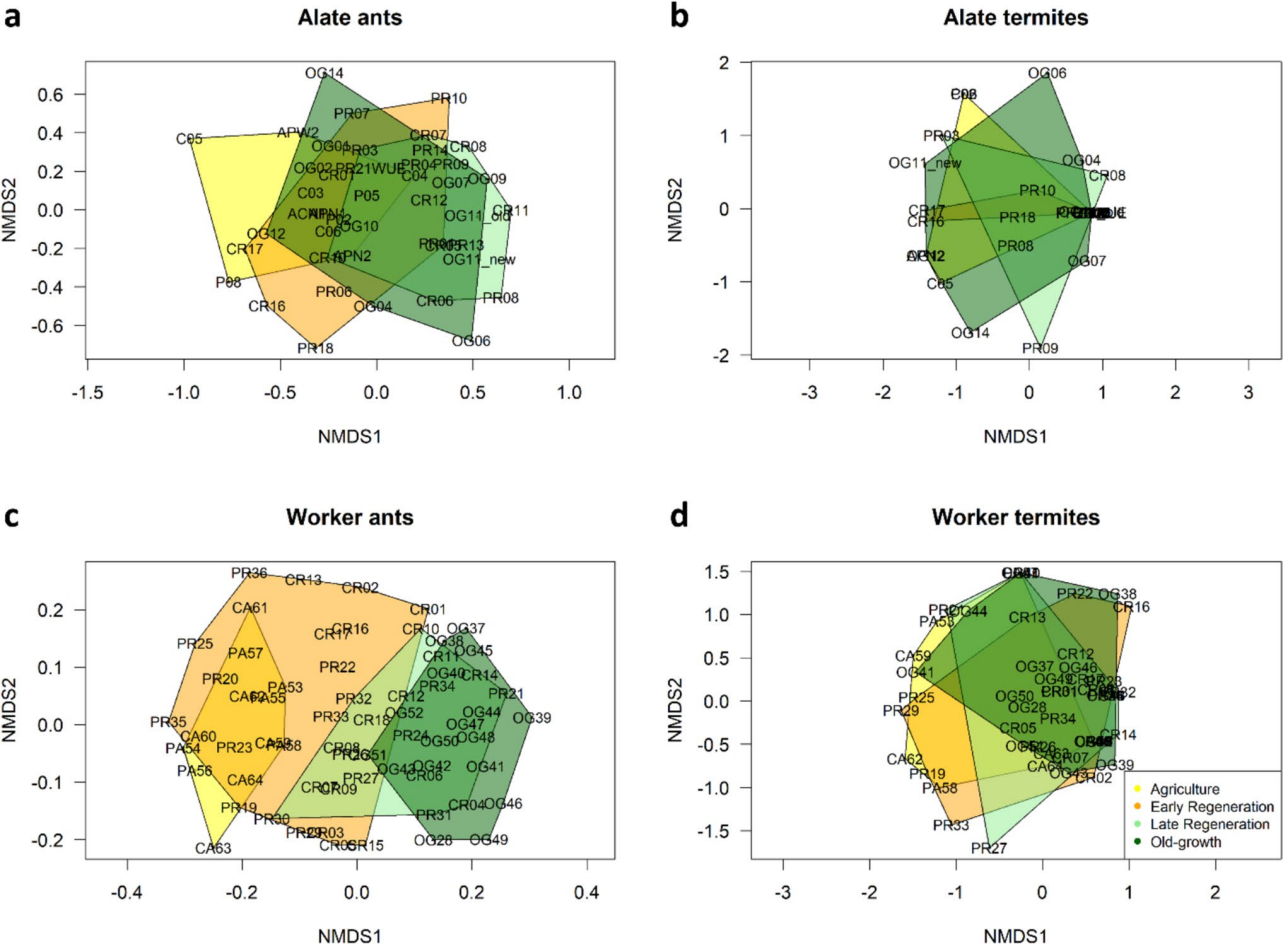
Non-metric multidimensional scaling (NMDS) of alate (**a, b**) and worker (**c, d**) assemblages of ants (**a, c**) and termites (**b, d**) in agricultural land, early regenerating forests (1–20 years), late regenerating forests (21–37 years), and old-growth forests

While the diversity can only give information about the number of OTUs in the plots, species composition considers the identities of observed taxa among plots. Our results from the analysis of alate assemblages of both taxa showed a strong overlap between agricultural land, regenerating forests, and old-growth forests (Fig. 3a,b) and the multiple regression models likewise showed that species composition (Jaccard dissimilarity index) is independent of forest age or elevation (Table 2). However, alate communities of both taxa were driven by space where communities were more similar in spatially closer plots (Table 2, Figure S3 a, b).

**Table 2:** Multiple regression models of alate and worker species composition (Jaccard dissimilarity index) of ants and termites with Jaccard dissimilarity, forest age, elevation, and space (longitude and latitude) as input dissimilarity matrices. Significant p values are highlighted in bold

	Estimate	*p* value
Ant alates		
Intercept	0.14	**0.001**
Forest age	0.03	0.14
Elevation	-0.03	0.18
Space	0.25	**0.001**
Ant workers		
Intercept	0.1	**0.001**
Forest age	0.14	**0.001**
Elevation	0.07	**0.001**
Space	0.002	0.82
Termite alates		
Intercept	0.09	0.9
Forest age	0.01	0.8
Elevation	-0.03	0.48
Space	0.15	**0.005**
Termite workers		
Intercept	0.11	0.36
Forest age	0.02	0.45
Elevation	0.12	**0.003**
Space	-0.06	**0.03**

Ant worker communities were best predicted by forest age and elevation with more similar communities on plots with similar forest ages and similar elevations (Fig. 3c, Figure S3e, Table 2). Termite worker assemblages were similar in all regeneration stages (Fig. 3d). However, communities were influenced by space and elevation with communities being more similar on similar elevations and more dissimilar when plots were spatially closer (Table 2, Figure S3d, Figure S3f).

## Discussion

### Ant and termite alates

The results of our species composition analysis of alates and workers deliver insights into ant and termite community assembly mechanisms in regenerating forests. Assemblages of dispersing alates of both taxa were more similar in spatially closer plots, while forest age and thus habitat type had no influence. This pattern indicates that alates from individual colonies likely do not disperse throughout the entire study area, resulting in spatially structured communities. These results align with our first scenario, where dispersal limitation operates at the landscape scale through spatial distance, but alates are not prevented from reaching different forest ages. The absence of forest age effects on alate communities suggests that habitat filtering associated with regeneration stage does not operate during the dispersal phase for either taxon.

However, this landscape-scale dispersal limitation should not prevent alates from reaching different habitat types. Our study area is characterized by a small-scale mosaic of habitat types, where agricultural plots and plots of different forest ages are distributed across the landscape. Although the study area spans over 200 km^2^, plots of different habitat types are interspersed, with minimum distances between plots of different types as short as 250 m and average inter-plot distances ranging from 4.6 to 5.5 km depending on habitat type (Escobar et al. 2025). This landscape configuration means that even species with limited dispersal ranges can potentially reach multiple habitat types from any given source colony.

Studies on flight ecology of these social insects are scarce, and dispersal distances are only known for a selected number of species. Ants have been shown to have a broad range of dispersal distances (see overview in Helms 2018). For example, for *Pheidole minutula*, a maximum dispersal distance of 30 m has been estimated (Bruna et al. 2011), for *Azteca* species up to 400 m (Bruna et al. 2011), *Atta sexdens* is estimated to disperse up to 11 km (Jutsum & Quinlan 1978), and *Solenopsis invicta* between 5.4 km and 32 km depending on the study (Helms 2018). For termites, that are typically regarded as poor fliers, there has been remarkably little research on flight distances, and most studies focused on pest species. The globally significant pest species *Coptotermes formosanus*, for example, has been shown to disperse mainly in a 250-m radius from the nest with some individuals dispersing as far as 1.3 km (Mullins et al. 2015), while another pest species, *Odontotermes formosanus*, has been shown to fly on average 146 m from its nest (Hu et al. 2010).

Given the dispersal distances reported in the literature and the small-scale landscape heterogeneity in our study area, ants and termites are likely capable of dispersing into plots of all forest ages where they could potentially establish new colonies. This accessibility across habitat types may explain why habitat type and thus forest age did not influence alate species composition. The spatial structure we observed therefore reflects the cumulative dispersal patterns of multiple colonies distributed across the landscape, rather than indicating that any particular habitat type is inaccessible to dispersing alates. While individual colonies may have limited dispersal ranges that may create spatial autocorrelation in our data, the landscape’s fine-scale mosaic structure likely ensures that alates from nearby colonies can reach sites representing all regeneration stages. Nevertheless, the spatial structure in alate communities demonstrates that dispersal limitation still operates at the landscape scale, with greater similarity among nearby plots—irrespective of forest age. This suggests that not all alates disperse across the full extent of our study area.

In addition, we recognize that our approach only indirectly investigates dispersal limitation based on spatial patterns between plots. Thus, we cannot exclude the possibility that environmental gradients correlated with spatial distance, rather than dispersal limitation per se, drive the observed spatial structure in alate communities. In general, little is known about flight behavior, habitat selection during nuptial flights, or how landscape features influence alate movement. Hence, for the understanding of alate dispersal, further insights from direct evidence would be needed such as marking and tracking individual alates, genetic analyses of kinship between colonies and alates, or experimental manipulations (e.g., Türke et al. 2010; Hu et al. 2010; Chen & Robinson 2013; Mullins et al. 2015).

### Ant workers

The results of the ant worker diversity analysis support previous findings where ant richness or diversity increases with forest age (Bihn et al. 2008; Rocha-Ortega and García-Martínez 2018; Karolak & Fiedler 2024). In contrast to ant alates, forest age and elevation influenced species composition with more similar OTUs being found in plots of similar forest ages and elevations. Although our elevation gradient only comprises 487 m, effects of elevation that are probably correlated with environmental conditions already affected species composition. This has been shown for ants globally (Smith 2015; Szewczyk and McCain 2016) but also in tropical and subtropical elevational gradients (Staab et al. 2014; Hethcoat et al. 2019; Leahy et al. 2024) and in our study area (Hoenle et al. 2022). Shifts in ant assemblages with regeneration time have also been extensively documented across multiple studies elsewhere (Dunn 2004; Bihn et al. 2008; Neves et al. 2010; Schmidt et al. 2013; Staab et al. 2014; Gomes et al. 2014; Hethcoat et al. 2019) and in the study region (Hoenle et al. 2022). Different forest ages represent different environments as environmental parameters change over time with succession. Two important abiotic parameters are temperature and humidity, which are linked to canopy openness. Logging and habitat conversion can alter the canopy openness resulting in hotter and drier below-canopy microclimates in comparison to forests. The maximum temperature in our study area has been shown to be 6.2° C lower in old-growth forests than in agriculture (Newell et al. 2025). For ants, canopy openness has been identified as a key driver of community dynamics in disturbed habitats (Andersen 2019). In Borneo, for example, Boyle et al. (2021) have shown that ants with higher thermal tolerance were more abundant in more disturbed and warmer habitats. Klimes et al. (2012) additionally identified tree density, tree size, and taxonomic diversity of trees as parameters explaining differences in ant communities during succession. As tree species richness and many structural variables such as light availability, maximum tree height, number of stems, vertical vegetation heterogeneity, aboveground biomass, diversity of coarse woody debris, and availability of fine woody debris have been shown to change with forest regeneration age in our study area (Falconí-López et al. 2024; Escobar et al. 2025), we argue that habitat filters associated with regeneration age might explain the observed distribution of ant workers. A previous analysis of regeneration mechanisms of our worker ant dataset has shown that after perturbation, ant regeneration is driven by the resistance of the species community rather than resilience (Metz et al., under review). This suggests that species composition along the regeneration gradient is rather driven by the persistence of ants remaining on or close to disturbed or previously disturbed land rather than by recolonization of arriving alates. Our results for ant workers correspond to the second scenario we outlined in the introduction, where alates disperse successfully across forest ages but worker communities are strongly filtered by forest age and elevation. This pattern indicates that habitat filtering operates primarily after dispersal, during colony establishment or persistence. The microhabitat requirements for successful colony foundation and growth, such as suitable nesting sites, stable microclimatic conditions, and adequate food resources, appear to vary substantially across the regeneration gradient. While we demonstrated that ant alates disperse into all forest regeneration ages, it remains uncertain whether the observed pattern of ant workers results from alates choosing or rejecting certain habitats for colony founding by the mated queens, or from the failure of the establishment of colonies that subsequently cannot persist in these environments. Additional research examining nesting site selection and post-establishment colony success rates would be valuable for further understanding the mechanisms of ant community reassembly.

### Termite workers

The results for termites were more complex and did not align with our expectations. As termites are considered to be sensitive to land-use changes (Ackerman et al. 2009; de Paula et al. 2016; Duran-Bautista et al. 2020, 2024; Castro et al. 2021), we expected differences in diversity and species composition depending on forest age. However, only the diversity of termite workers was influenced by forest age in our study, while species composition was influenced by elevation and showed unexpectedly greater dissimilarity in spatially closer plots. Although termites generally build their nests in different forest strata such as soil, forest floor, or on trees, termite workers were mostly found in the additionally sampled naturally occurring dead wood or in dead wood baits in our study. A former study on the same plots showed that the amount of coarse woody debris did not differ with forest age (Falconí-López et al. 2024), while other habitat variables such as forest structural complexity (Escobar et al. 2025) and tree-related microhabitats such as tree rot-holes and dead lianas (Hausmann et al. 2026) did increase with forest age. Hence, we suggest that the increased availability and diversity of dead wood substrates could provide more resources for promoting a higher species diversity, but that the species assemblages are homogeneous across all forest ages as dead wood-dwelling termites might be protected from disturbance such as increased heat exposure.

Despite more similar alate communities in spatially closer plots, termite workers showed the opposite pattern with more dissimilar communities in spatially close plots. We hypothesize that this could be caused by competition for dead wood as a food or nesting resource. In general, termites have a specialized dietary niche comprising predominantly dead plant material at various stages of decomposition (Donovan et al. 2001). With six of 11 collected genera, most of our collected termites belonged to wood-feeding guilds, compared to four soil-feeding genera and one litter-feeding genus based on the trophic classifications by Donovan et al. (2001). Competition for food or resources is expressed in agonistic behavior (Prestwich 1984; Thorne & Haverty 1991; Shelton & Grace 1996; Šobotník et al. 2010). Termites from the frequently observed genus *Nasutitermes* have been shown to exhibit intra- and interspecific agonistic behavior by defending foraging areas in natural inter-colony encounters (Levings & Adams 1984), in field manipulation induced intraspecific encounters, or in laboratory experiments (Thorne 1982; Leponce et al. 1996). Another study on *Anoplotermes banksi* delivered evidence that nest distribution may be driven by intraspecific competition resulting in overdispersion of nests of the same species (Bourguignon et al. 2011). However, the influence of interspecific interactions on species assembly is difficult to observe or measure. With our methodological approach, we only indirectly measured assemblage dissimilarities caused by spatial proximity. In addition, other forest parameters that were not accounted for could result in the observed pattern. Hence, further research is needed to get a deeper understanding of the spatial distributions of termite communities and the role of species interactions in community assembly.

In addition to the effect of space, we also found an effect of elevation on termite worker assemblages. This has frequently been observed before (Gathorne-Hardy et al. 2001; Palin et al. 2011; Nunes et al. 2017) and could be driven by elevation-associated habitat filters such as differences in microclimate. For instance, along a neotropical elevation gradient ranging from 800 to 1400 m, Nunes et al. (2017) have shown a species turnover with elevation. As the same functional groups were present at different elevations but consisted of different species, they suggested an environmental filtering for physiological adaptations to elevation.

Hence, from our results we conclude that the sampled worker termite communities might not be driven by habitat filters associated with forest age, but by habitat filters associated with elevation as well as by spatial effects. As in our study termite diversity was not affected by elevation, we suggest that habitat filters associated with elevation might only filter for specific functional guilds but does not influence the overall diversity.

### Limitations

Several limitations should be considered when interpreting our results. For instance, our alate and worker datasets necessarily represent different portions of the regional species pool rather than dispersing and established stages of identical communities. This difference arises from both biological and methodological factors. Biologically, the alate dataset captures only species that disperse via nuptial flights with winged reproductives, while the worker dataset additionally includes species employing alternative dispersal mechanisms such as colony fission, budding, or wingless queen dispersal (Peeters & Ito 2001). Furthermore, nuptial flights and colony activity undergo seasonal fluctuations (Basset et al. 2023), meaning that the temporal snapshot captured by our sampling may not represent the same seasonal communities for both life stages.

Methodologically, we employed different sampling approaches for the two datasets. Worker sampling targeted species nesting and foraging in lower forest strata (soil, leaf litter, forest floor, dead wood, tree trunks) using multiple methods across multiple sampling events, while alate sampling used single night light trap deployments per plot. Hence, light traps could attract arboreal species that are underrepresented in worker sampling, potentially capturing a broader vertical stratification of the assemblages. In addition, light traps may actively attract alates rather than passively intercepting natural dispersal flows, and their detection radius likely varies with forest structure. Open habitats such as pastures and young secondary forests allow light to travel farther than closed canopy forests, which could theoretically introduce sampling bias across the forest age gradient.

However, these limitations do not undermine our main conclusions for several reasons. First, we applied each sampling methodology uniformly across all plots, ensuring that we sampled comparable subsets of the regional species pool at each site. This uniform approach makes spatial and environmental comparisons valid even though our samples do not capture the complete regional species pool as our study focused on comparing assemblages across habitat types and spatial distances rather than documenting complete inventories. Second, by analyzing alate and worker communities as separate datasets, we independently assess dispersal limitation and habitat filtering for each life stage. This approach allows us to draw conclusions specific to each sampled assemblage without requiring that both datasets represent identical species. Third, regarding the potential detection bias from light traps, this would be expected to bias our results toward finding stronger forest age effects on alate communities because open habitats would be oversampled. Yet, we observed the opposite pattern, with alate communities showing remarkable similarity across the forest age gradient. The absence of forest age effects on alate communities despite this potential detection bias strengthens our conclusion that alates can access habitats across the regeneration gradient.

The alates we captured represent species that were dispersing simultaneously across the landscape during our sampling window. The absence of dispersal limitation in this temporally defined assemblage is ecologically meaningful, as it demonstrates that alates engaged in concurrent dispersal events successfully reached sites across all habitat types and regeneration ages. This indicates that dispersal ability does not limit species assembly for this co-dispersing assemblage across the different habitats in our study area. Similarly, the worker communities we sampled represent established colonies that were active and foraging at the time of sampling. Although neither dataset captures the complete regional species pool, they provide valuable insights into the assembly processes operating at different life stages for the communities we were able to sample.

## Conclusion

By analyzing the species composition of dispersing alates as well as workers originating from established colonies, we successfully disentangled the relevance of dispersal and habitat filters for ants and termites along a regeneration gradient of a lowland tropical rainforest. Our results showed that alate communities of ants and termites are similar along the regeneration gradient. This indicates that they might not avoid or prefer certain forest types during their nuptial flights and that they have the potential to reach and colonize forests of all ages. As alate communities were more similar in spatially closer plots, we conclude that their dispersal distances might be smaller than the spatial extent of our study area.

In contrast, the worker communities were driven by forest age and elevation for ants, and elevation and space for termites. This indicates that habitat features attributed to forest age, elevation, or possibly interspecific interactions (in the case of termites) could influence the foundation and/or persistence of colonies of these social insects. From these results, we conclude that habitat filtering might be a more important driver for the distribution of colonies than an initial dispersal limitation by alates in the context of forest regeneration.

Our findings have important implications for landscape-scale forest management and restoration in the Chocó–Darién biodiversity hotspot and similar tropical ecosystems. Since alates successfully disperse across the forest age gradient within our study area, dispersal limitation appears less critical than habitat filtering for structuring ant and termite communities during forest regeneration. Providing sufficient areas of old-growth forest and older regenerating forests is particularly important, as these habitat types supported the highest species diversities for both taxa in our study. The habitat conditions associated with advanced forest succession, such as complex forest structure, dead wood resources, and stable microclimatic conditions, appear essential for maintaining diverse species assemblages. Similarly, the strong elevation effects we observed for both ants and termites suggest that topographic heterogeneity creates distinct habitat conditions that support different communities, emphasizing the need to protect forests across elevational gradients.

However, our conclusions about dispersal limitation apply specifically to species that disperse via nuptial flights. Species relying primarily on colony fission or budding are expected to have much shorter dispersal distances and were probably underrepresented in our alate sampling. These short-distance dispersers would likely face stronger dispersal limitation and may benefit more from reduced distances between suitable habitat patches. Future conservation planning should therefore consider the full spectrum of dispersal strategies present in ant and termite communities.

More broadly, our study demonstrates that dispersal limitation and habitat filtering can operate at different spatial scales and life stages within the same taxonomic groups. The decoupling of dispersal (alate stage) from establishment and persistence (colony stage) in social insects provides a clear example of how focusing solely on dispersal ability may underestimate the importance of post-dispersal filters in structuring communities. In the context of ongoing deforestation and land-use transformation, our results suggest that regenerating secondary forests can be reached by dispersing social insects from remaining forest patches. However, whether these regenerating forests can support the full diversity of ant and termite colonies depends critically on the trajectory and pace of forest recovery, as habitat conditions associated with forest age and elevation determine colony establishment and persistence. Given the increasing reliance on secondary forests for biodiversity conservation (Chazdon et al. 2009; Chazdon & Guariguata 2016) and the complexity of regeneration processes (Poorter et al. 2021), understanding these assembly mechanisms is crucial for developing evidence-based management strategies that promote the recovery of functionally diverse social insect communities.

## Supplementary Information

Below is the link to the electronic supplementary material.

Supplementary file1 (DOCX 686 KB)

## Data Availability

Annotated R code, including the data needed to reproduce the statistical analyses and figures as well as supporting information, is publicly available from figshare: 10.6084/m9.figshare.28650614.v1.
