# Supplementary material for: Habitat filtering, not dispersal limitation, drives ant and termite community assembly along a tropical forest regeneration gradient
Source: Oecologia. 2026 Mar 15;208(3):43. doi: 10.1007/s00442-026-05875-9 (PMC12992394; doi:10.1007/s00442-026-05875-9)
Supplement: Supplementary file 1 — Supplementary file1 (DOCX 686 KB) [file 442_2026_5875_MOESM1_ESM.docx]

**Supplementary Material**

**Habitat filtering, not dispersal limitation, drives ant and termite community assembly along a tropical forest regeneration gradient**

Nina Grella, David A. Donoso, Jörg Müller, Ana Falconí-López, Annika Busse, Peter Kriegel, Marcel Püls, Dominik Rabl, Sebastian Seibold, Heike Feldhaar


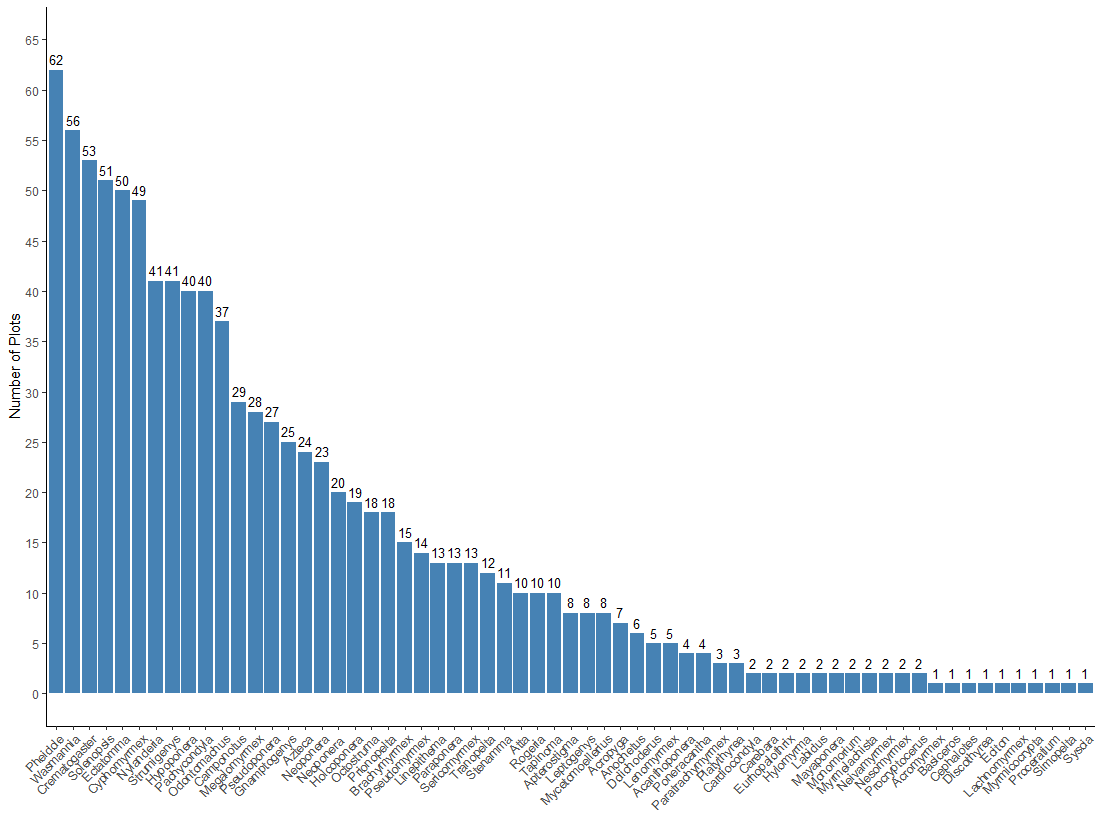


**Figure S1**: Number of sampling plots where each ant genus was detected. Only data from the collection of workers are included. The values above each bar represent the total count of plots where specimens from each genus were collected.


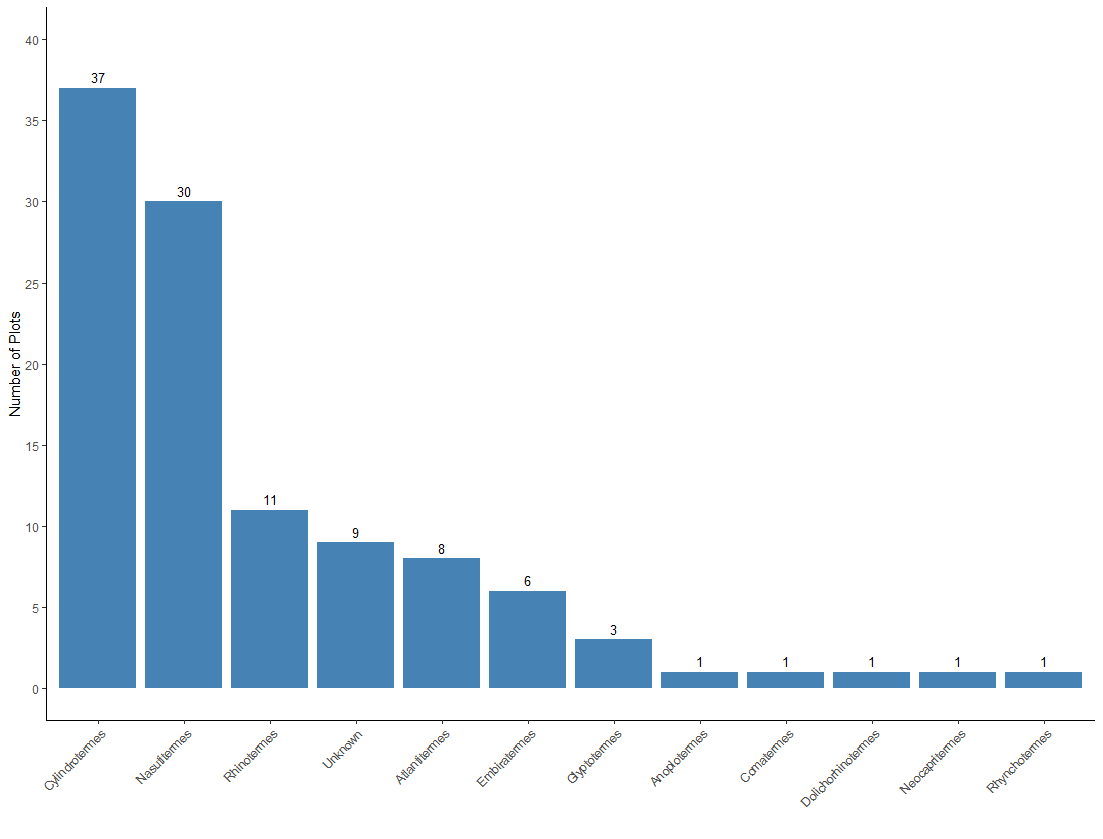


**Figure S2:** Number of sampling plots where each termite genus was detected. Only data from the collection of workers are included. The values above each bar represent the total count of plots where specimens from each genus were collected.


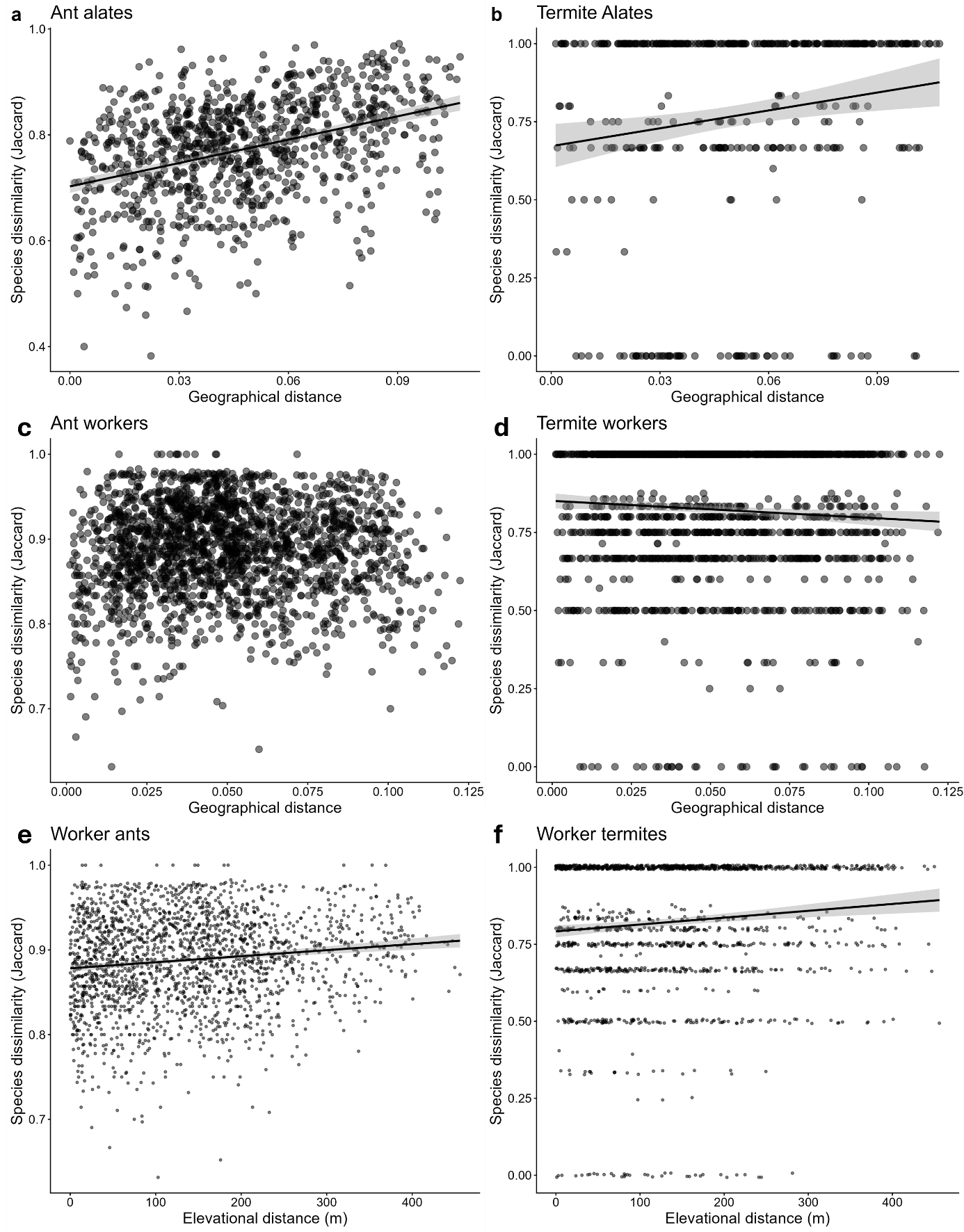


**Figure S3**: Species dissimilarity of ant (a,c,e) and termite (b,d,f) alates (a,b) and workers (c,d,e,f) by geographical distances of the plots (a-d) and by elevational distances of the plots (e, f). Their trajectories are predicted by a linear model with 95% confidence interval. Solid lines indicate a significant trend of species dissimilarity according to generalized linear models.
